# Bariatric Surgery Improves Cardiac Remodeling and Function in Adults With Preserved Left Ventricular Ejection Fraction

**DOI:** 10.1016/j.jacadv.2026.103040

**Published:** 2026-08-26

**Authors:** Marie-Eve Piché, Géraldine Ong, Caroline Samhani, Fannie Lajeunesse-Trempe, Audrey Auclair, Philippe Pibarot, Paul Poirier

**Affiliations:** a Institut Universitaire de Cardiologie et de Pneumologie de Québec-Université Laval, Québec, Québec, Canada; b Faculty of Medicine, Laval University, Québec, Québec, Canada; c Division of Cardiology, St. Michael’s Hospital, University of Toronto, Toronto, Ontario, Canada; d Faculty of Pharmacy, Laval University, Québec, Québec, Canada

**Keywords:** bariatric surgery, cardiac function, cardiac remodeling, heart failure, obesity, visceral adiposity

## Abstract

**Background:**

Obesity is a risk factor for heart failure. Surgical treatment of obesity may help prevent heart failure.

**Objectives:**

We examine the effect of bariatric surgery on cardiac remodeling and function in adults with preserved left ventricular (LV) ejection fraction.

**Methods:**

Thirty-eight individuals with severe obesity (body mass index ≥35 kg/m^2^) and preserved LV ejection fraction (≥50%) who underwent bariatric surgery (biliopancreatic diversion with duodenal switch), and 34 individuals with obesity managed medically were included. Conventional echocardiography and speckle tracking echocardiography were obtained at baseline and 12-month follow-up.

**Results:**

The mean age of participants (75% female) was 48.5 ± 8.3 years and body mass index 46.4 ± 3.7 kg/m^2^. Bariatric surgery led to marked weight reduction (percentage of total weight loss 36.7% ± 7.1%) and significant improvements in obesity-related metabolic conditions/comorbidities. Bariatric surgery was associated with a significant reduction in LV mass and LV hypertrophy (*P* < 0.001), improvement in LV diastolic function parameters (*P* < 0.01), and improvement in subclinical systolic function (LV global longitudinal strain, 16.3% ± 2.5% vs 18.5% ± 1.3%, *P* < 0.05). Preoperative abnormalities of subclinical cardiac function were highly prevalent (79%) and normalized in 70% of subjects after surgery. Subjects with obesity managed medically remain with a significant burden of comorbidities and a number (38%) worsened their subclinical systolic function during the follow-up (*P* < 0.05).

**Conclusions:**

Bariatric surgery may be an effective and safe way to prevent and/or reverse obesity-related cardiac abnormalities. Larger prospective studies are warranted to confirm these observations and determine their long-term clinical implications.

Obesity (body mass index [BMI] ≥30 kg/m^2^) is a major risk factor for heart failure (HF), particularly HF with preserved ejection fraction.^1^^,^^2^ One-third of adults with obesity show clinical evidence of HF, and the probability of HF increases with obesity severity and duration.^3^ Obesity and coexisting metabolic conditions/comorbidity such as hypertension, dysglycemia, and insulin resistance, predispose to structural and functional cardiac abnormalities that may be encountered in the asymptomatic phase (ie, subclinical cardiac disease) of cardiac involvement.^4^ The prevalence of asymptomatic alterations of cardiac function in obesity has been reported to range from 37% to 54%.^5^ These features confers an increased cardiovascular risk, and may predispose to the development of HF.^6^

Bariatric surgery has been proven the most effective treatment to induce durable weight loss and reverses multiple underlying obesity-related metabolic conditions/comorbidities involved in HF.^7^^,^^8^ We previously showed that bariatric surgery improves cardiac structure and function early after surgery in subjects with preserved left ventricular (LV) ejection fraction.^9^ In a study of adults with obesity, bariatric surgery was associated with half less incidence of HF compared with intensive lifestyle treatment of obesity.^10^ In a long-term follow-up of the Swedish Obesity Surgery study, bariatric surgery lower risk of developing HF (35% reduction) compared to usual medical care.^11^ Among bariatric surgery procedures, biliopancreatic diversion with duodenal switch (BPD-DS) is the most effective at inducing sustained weight loss and metabolic recovery.^12^ No study has studied the long-term effect of BPD-DS on cardiac remodeling and function including LV and right ventricular (RV) strain, and using a comprehensive assessment of metabolic markers including visceral adiposity in adults without known cardiac disease. We investigated the effects of bariatric surgery on cardiac remodeling and function in adults with preserved LV ejection fraction.

## Material and methods

### Participants

A total of 104 participants, aged between 18 and 65 years, with criteria for bariatric surgery (BMI ≥40 kg/m^2^ or ≥35 kg/m^2^ with associated comorbidities)^13^ were prospectively recruited through the bariatric surgery clinic of the Institut de cardiologie et de pneumologie de Québec – Université Laval. Those with a past medical history of cardiovascular diseases or LV ejection fraction <50%, as well as those with technically limited echocardiograms and poor image quality rejected by the software were excluded. Participants fulfilling all of the aforementioned criteria (n = 68) were included in the study. From these participants, 38 underwent BPD-DS (surgical group). The BPD-DS procedure consisted of a sleeve gastrectomy and an ileal anastomosis 250 cm from the ileocecal valve with a common limb of 100 cm.^14^ Consecutive patients with BMI ≥40 kg/m^2^ treated medically followed in the Bariatric Clinic were included. For the medical group, standard of care was strongly emphasized.^15^ Each patient was supported by a specialized obesity team consisting of a nurse clinician, dietitian, kinesiologist, and psychologist. Management involved personalized nutritional counseling, structured lifestyle programs, prescribed exercise, and behavioral therapy, with ongoing follow-up to ensure adherence and monitor outcomes. Anthropometric, biochemical, and echocardiographic measurements were performed at baseline and at 12-month follow-up. The study protocol was approved by the Ethics Committee of the Institut de cardiologie et de pneumologie de Québec-Université Laval. All participants gave written informed consent.

### Metabolic conditions/comorbidities definitions

Type 2 diabetes status was determined from patient’s biochemistry and medical record in accordance with the American Diabetes Association guidelines.^16^ According to the American Diabetes Association consensus statement, type 2 diabetes remission was defined as having fasting blood glucose <5.6 mmol/L and/or glycosylated hemoglobin ≤6.0% in the absence of glucose-lowering pharmacological therapy.^17^ Hypertension status was determined from the patient’s medical record and resolution was defined by the absence of antihypertensive medication and with a resting blood pressure <140/90 mm Hg.

### Biochemical analysis

N-Terminal pro–B-type natriuretic peptide (NT-proBNP) concentrations were measured with a Modular system from Roche Diagnostics and Roche reactants (Roche). Plasma cholesterol, triglycerides, and lipoprotein fractions concentrations were determined in plasma using a Technicon RA-500 analyzer (Bayer Corporation).^18^ High-sensitivity C- reactive protein levels were obtained by immunoassay (Dade Behring). Insulin sensitivity was assessed with the homeostasis assessment of insulin resistance model.^19^

### Anthropometric measurements

Body weight, fat mass, and fat free mass were assessed using a bioelectrical impedance balance (Tanita TBF-350). Abdominal visceral adipose tissue levels were assessed with computed tomography (CT) scan.^20^ CT-scan images were analyzed using specialized image analysis software (Tomovision SliceOMatic 4.3 Rev-6f software) as previously described.^20^

### Heart rate variability

Heart rate variability was measured using a 24-hour Holter monitoring system (Marquette Electronics Inc.). As described previously,^21^ parasympathetic activity has been assessed with time-domain parameters: square root of the mean squared difference of successive RR intervals, percentage of differences between adjacent normal RR intervals exceeding 50 milliseconds and high frequency parameters. Sympatho-vagal balance was defined as the ratio of low frequency/high frequency.

### Echocardiographic measurements

All participants underwent a transthoracic echocardiographic examination following the recommendations of the American Society of Echocardiography/European Association of Echocardiography recommendations.^22^ LV ejection fraction was measured with the use of the biplane Simpson method. LV mass was calculated according to guidelines^22^ and further indexed to a 2.7 power of height (left ventricular mass index [LVMi]^2^^.7^).^23^ LV hypertrophy was defined as LVMi^2.7^ >49 g/m^2.7^ in men and >47 g/m^2.7^ in women.^24^ By taking into account both values of LVMi^2.7^ and relative wall thickness ratio, patients were classified into 4 LV remodeling patterns using criteria proposed by the American Society of Echocardiography and the European Association of Cardiovascular Imaging (ASE–EACVI).^25^ Patients were also classified according to LV diastolic function grade.^26^ LV global longitudinal strain (GLS) was retrospectively measured from 2D echocardiography image acquisition (GE Vivid 95i or Philips [EPIC 7]) with the Velocity Vector Imaging program using a commercially validated software (Leiden University Medical Center: EchoPac, version 113; 2D Cardiac Performance Analysis; TomTec Imaging Systems). LV GLS was calculated by averaging the value of 18 segments from the 4-chamber, 3-chamber, and 2-chamber apical views.^27^ Two observers blinded to the clinical information performed all GLS measurements. Our interobserver and intraobserver variabilities in measurement of GLS have been reported.^28^ A LV GLS of <18% was considered abnormal, corresponding to the average minus 2 SD of a nonobese control group.^25^

### Statistical analysis

Continuous variables were reported as mean and SD. Categorical variables were described as frequencies and percentages. Distribution of data was verified using normality tests (Shapiro-Wilk, Kolmogorov-Smirnov), skewness, and kurtosis statistics. Differences between groups were compared by Student’s *t*-test with homogeneity of variances assessed via Levene test for equality of variance. Categorical data and proportions were analyzed using the chi-square test, the Fisher exact test, and the McNemar test, as appropriate. Repeated-measurements analysis of variance was used to compare dependent variables (ie, GLS and metabolic conditions/comorbidities) between groups (surgical vs medical) across time. Post hoc comparisons between groups, when necessary were performed by using the Holm-Sidak post hoc test. All models included group and time as independent variables as well as the interaction of group and time. Group × time interaction was used to establish differences in response to the intervention (surgical vs medical) between groups. Pearson or Spearman correlations, as appropriate, were used to detect univariate associations between baseline and delta changes after the bariatric surgery procedure. Multiple stepwise linear regression analyses were additionally performed to assess the independent predictors of subclinical cardiac function (LV GLS) change consecutive to bariatric surgery intervention. The variables that were entered in the model were those with clinical relevance and/or a *P* value < 0.05 after univariate analysis. Two-sided *P* values < 0.05 were considered statistically significant. Data were analyzed using SAS software version 9.4 (SAS Institute Inc).

## Results

### Participant clinical characteristics

**Figure 1:**
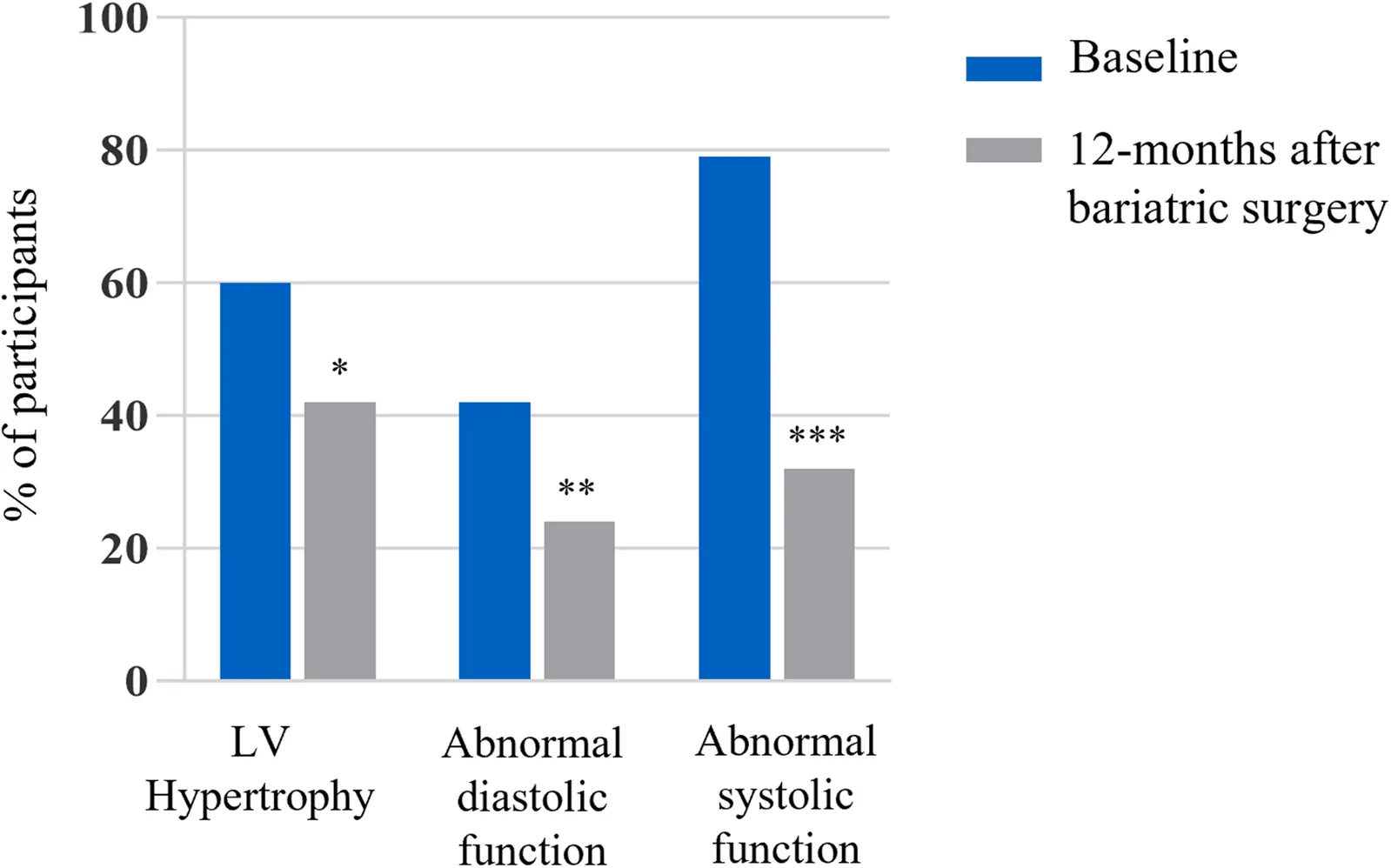
Changes in Cardiac Remodeling and Function Following Bariatric Surgery Graph bars represent the proportion of adults with preoperative (blue) and postoperative (gray). (A) Left ventricular hypertrophy, (B) abnormal diastolic function, and (C) abnormal systolic function (abnormal [left ventricular global longitudinal strain <18%]). ∗*P* < 0.05, ∗∗ <0.01, ∗∗∗<0.001. LV = left ventricular.

Thirty-eight patients with severe obesity (mean BMI: 48 ± 7 kg/m^2^; 89% women) who underwent bariatric surgery and 34 patients with obesity (mean BMI: 44 ± 3 kg/m^2^; 60% women) on medical treatment were included. In the surgical group, 40% had type 2 diabetes, 61% hypertension and 63% sleep apnea (Table 1). LV dimensions and LV ejection fraction of participants were within normal limits (Table 2). Preoperative abnormalities of LV diastolic function were present in 42% of surgical participants. The preoperative subclinical cardiac function was reduced (LV GLS: 16.3% ± 2.5%); 79% of surgical participants have abnormalities of subclinical cardiac function (LV GLS <18) (Figure 1).

**Table 1:** Participant Characteristics

	Surgical Group		Medical Group	
	Baseline	12 Months	Baseline	12 Months
Age, y	41 ± 11	-	56 ± 14^b^	-
Female, n (%)	34 (89)	-	20 (60)	
BMI, kg/m^2^	48.4 ± 7.4	30.6 ± 5.7^a^	44.4 ± 2.9	43.2 ± 3.8^a^^,^^b^^,G,G∗T^
Visceral adipose tissue, cm^2^	279.7 ± 85.9	102.4 ± 55.7^a^	-	-
Resting heart rate, bpm	80 ± 11	62 ± 9^a^	77 ± 14	74 ± 12^b^^,G,G∗T^
Systolic blood pressure, mm Hg	130 ± 14	115 ± 12^a^	136 ± 18	136 ± 23^b^^,G,G∗T^
Diastolic blood pressure, mm Hg	81 ± 9	74 ± 9^a^	75 ± 15	75 ± 13
Comorbidities, n (%)				
Hypertension	23 (61)	11 (29)^a^	18 (53)	19 (56)
Type 2 diabetes	15 (40)	1 (3)^a^	12 (35)	12 (35)
Sleep apnea	24 (63)	4 (11)^a^	10 (29)	11 (32)
Medication, n (%)				
β-Blockers	5 (13)	3 (8)^a^	13 (38)	13 (38)
ACE inhibitor	9 (24)	2 (5)^a^	9 (26)	9 (26)
ARB	5 (13)	3 (8)^a^	3 (9)	3 (9)
Calcium channel blocker	7 (18)	4 (11)^a^	5 (15)	4 (12)
Diuretics	9 (24)	3 (8)^a^	12 (35)	15 (44)
Metabolic biomarkers				
Fasting glucose, mmol/L	6.7 ± 2.5	4.8 ± 0.4^a^	6.7 ± 2.4	6.3 ± 1.3^b^^,G,G∗T^
HOMA-IR index	7.0 ± 5.3	1.1 ± 0.7^a^	-	-
HbA1c, %	6.1 ± 1.1	5.1 ± 0.4^a^	6.5 ± 0.2	6.2 ± 0.1^b^^,G,G∗T^
Creatinine levels, mg/dL	68 ± 15	63 ± 16^a^	77 ± 6	78 ± 6
LDL cholesterol, mmol/L	2.7 ± 0.7	1.6 ± 0.4^a^	2.3 ± 0.9	2.3 ± 0.8^b^^,G,G∗T^
Non-HDL cholesterol, mmol/L	3.7 ± 1.2	2.8 ± 0.8^a^	3.0 ± 1.0	3.1 ± 0.9^b^^,G∗T^
HDL cholesterol, mmol/L	1.4 ± 0.3	1.2 ± 0.3^a^	1.1 ± 0.2	1.1 ± 0.2^b^^,G∗T^
Apolipoprotein B, g/L	0.78 ± 0.18	0.50 ± 0.12^a^	0.79 ± 0.08	0.79 ± 0.07^b^^,G,G∗T^
Triglycerides, mmol/L	1.6 ± 0.8	1.0 ± 0.4^a^	1.9 ± 0.3	1.7 ± 0.2^b^^,G∗T^
hs-CRP, mg/L	11.1 ± 9.2	1.7 ± 1.7^a^	8.5 ± 2.4	6.3 ± 1.7^b^^,G∗T^
Adiponectin μg/mL	5.9 ± 0.6	10.4 ± 1.6^a^	-	-
NT-proBNP, pg/mL	50 ± 7	147 ± 18^a^	288 ± 91	254 ± 57^b^^,G,G∗T^

Values are mean ± SD or n (%).

G, group effect; G∗T, time × group interaction.

ACE inhibitor = angiotensin converting enzyme inhibitor; ARB = angiotensin receptor antagonist; BMI = body mass index; HbA1c = glycosylated hemoglobin; HDL = high-density lipoprotein; HOMA-IR = homeostasis model assessment of insulin resistance; hs-CRP = high-sensitivity C- reactive protein; LDL= low-density lipoprotein; NT-proBNP = N-terminal pro–B-type natriuretic peptide.

a *P* < 0.01 baseline vs 12 months.

b *P* < 0.05 medical vs surgery (repeated measures).

**Table 2:** Echocardiographic Characteristics

	Surgical Group		Medical Group	
	Baseline	12 Months	Baseline	12 Months
Age, y	41 ± 11	-	56 ± 14^a^	-
BMI, kg/m^2^	48.4 ± 7.4	30.4 ± 6.1^b^	44.4 ± 2.9	43.2 ± 3.8^a^^,G,G^∗^T^
BSA, m^2^	2.2 ± 0.2	1.8 ± 0.2	2.3 ± 0.2	2.5 ± 0.2^a^^,G,G^∗^T^
IVS thickness, mm	10.9 ± 1.6	10.0 ± 1.7^b^	10.5 ± 1.9	11.1 ± 1.6^a^^,G^∗^T^
PW thickness, mm	9.5 ± 1.5	8.7 ± 1.4^b^	10.0 ± 1.9	10.6 ± 2.0^a^^,G,G^∗^T^
LVEDD, mm	49 ± 4	50 ± 4	49 ± 5	45 ± 5^a^^,G^
LVESD, mm	29 ± 4	30 ± 4	28 ± 4	32 ± 4^a^^,G^
LA diameter, mm	40 ± 3	39 ± 4^b^	35 ± 6	34 ± 8^a^^,G^
LV mass, g	184 ± 44	169 ± 47^b^	195 ± 44	198 ± 48^a^^,G,G^∗^T^
Indexed LV mass, g/m^2.7^	50.5 ± 9.4	46.4 ± 11.2^b^	49.9 ± 10.4	50.2 ± 11.1^a^^,G^∗^T^
LV ejection fraction, %	68 ± 7	63 ± 5^b^	66 ± 7	61 ± 5^a^^,G,G^∗^T^
RWT ratio	0.41 ± 0.18	0.35 ± 0.04^b^	0.41 ± 0.08	0.44 ± 0.09^a^^,G,G^∗^T^
Mitral E-wave velocity, cm/s	88 ± 17	89 ± 18	94 ± 21	87 ± 17
Mitral A-wave velocity, cm/s	76 ± 25	63 ± 22^b^	80 ± 17	84 ± 21^a^^,G,G^∗^T^
Mitral E/A ratio	1.25 ± 0.40	1.56 ± 0.53^b^	1.12 ± 0.44	1.08 ± 0.29^a^^,G,G^∗^T^
Mitral e’ lateral	8.3 ± 2.8	7.6 ± 2.5^b^	10.7 ± 3.6	10.6 ± 4.1^a^^,G,G^∗^T^
Mitral average E/e’	9.5 ± 2.8	8.7 ± 2.3^b^	9.8 ± 3.5	10.8 ± 4.0^a^^,G^∗^T^
Mitral DT, ms	196 ± 37	197 ± 35	192 ± 38	194 ± 45
LV global longitudinal strain, %	16.3 ± 2.4	18.5 ± 1.3^b^	20.2 ± 1.9	20.3 ± 1.9^a^^,G,G^∗^T^
RV free-wall strain (%)	22.4 ± 2.9	23.7 ± 3.6	22.4 ± 3.3	22.5 ± 2.7
RV global longitudinal strain, %	22.0 ± 2.7	22.5 ± 2.7	22.2 ± 3.2	22.5 ± 2.7
RV Tapse	2.4 ± 0.4	2.6 ± 0.3	2.2 ± 0.3	2.1 ± 0.4^a^^,G,G^∗^T^

Values are mean ± SD.

G, Group effect; G∗T, time × group interaction.

BMI = body mass index; BSA = body surface area; DT = deceleration time; IVS = interventricular septum; LA = left atrial; LV = left ventricular; LVEDD = left ventricular end-diastolic diameter; LVESD = left ventricular end-systolic diameter; PW = posterior wall; RWT = relative wall thickness; RV = right ventricular.

a *P* < 0.05 medical vs surgery (repeated measures).

b *P* < 0.05 baseline vs 12 months.

### Clinical and metabolic changes after bariatric surgery

The average BMI reduction during follow-up was 18 ± 6 kg/m^2^, and 47% of the initially severely obese individuals remained in the obesity range category (BMI ≥30 kg/m^2^) at 12 months. The percentage of total weight loss at 12 months after bariatric surgery was 36.7% ± 7.1%. Weight loss surgery was accompanied by a 63% reduction in visceral adipose tissue levels (280 ± 86 vs 102 ± 56 cm^2^, *P* < 0.001). Ninety-three percent of subjects achieved remission of type 2 diabetes. Glycosylated hemoglobin, insulin resistance, heart rate, blood pressure, lipid-lipoprotein levels, and inflammatory markers were all significantly improved following bariatric surgery (all *P* < 0.01). Forty-eight percent of participants on preoperative antihypertensive did no longer use this medication at 12-month follow-up.

### Structural changes and cardiac remodeling after bariatric surgery

**Figure 2:**
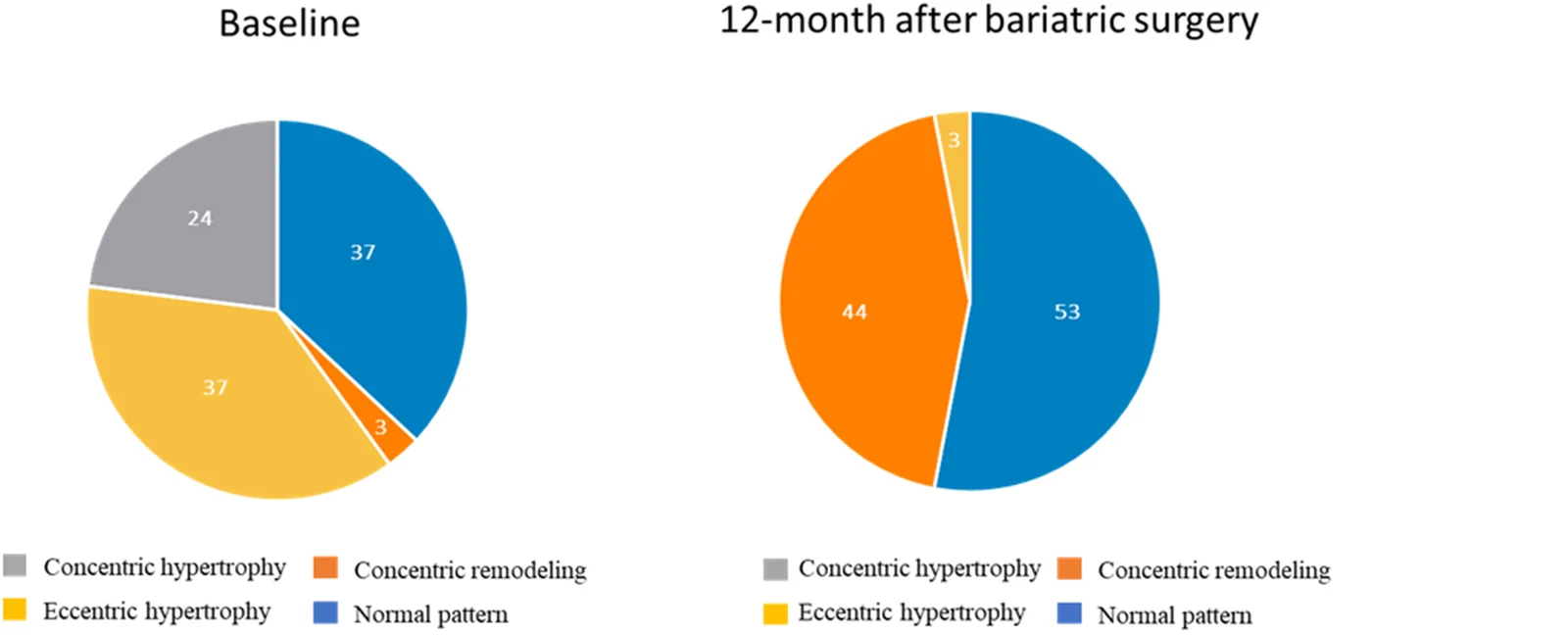
Changes in Patterns of Left Ventricular Remodeling After Bariatric Surgery Left ventricular remodeling was classified into 1 of 4 categories based on left ventricular mass index (left ventricular mass/height 2.7) and relative wall thickness. The numbers inside the bar are the actual corresponding percentage of individuals within each pattern. At baseline, eccentric hypertrophy was the most common pattern. After 12 months of follow-up, there was a shift toward less eccentric hypertrophy and more concentric remodeling.

Following bariatric surgery, LV wall thickness and LV mass significantly decreased (*P* < 0.05). The prevalence of LV hypertrophy at follow-up was reduced from 61% to 42%, most markedly among those with eccentric hypertrophy (Figure 1). Redistribution of cardiac remodeling patterns were observed (Figure 2). Resting heart rate decreased from 80 ± 11 to 62 ± 9 beats/min (*P* < 0.05) and systolic blood pressure was significantly reduced (130 ± 14 to 115 ± 12 mm Hg) (*P* < 0.05). Heart rate variability parameters also improved after bariatric surgery (Supplemental Table 1).

### Clinical and subclinical cardiac function change after bariatric surgery

**Figure 3:**
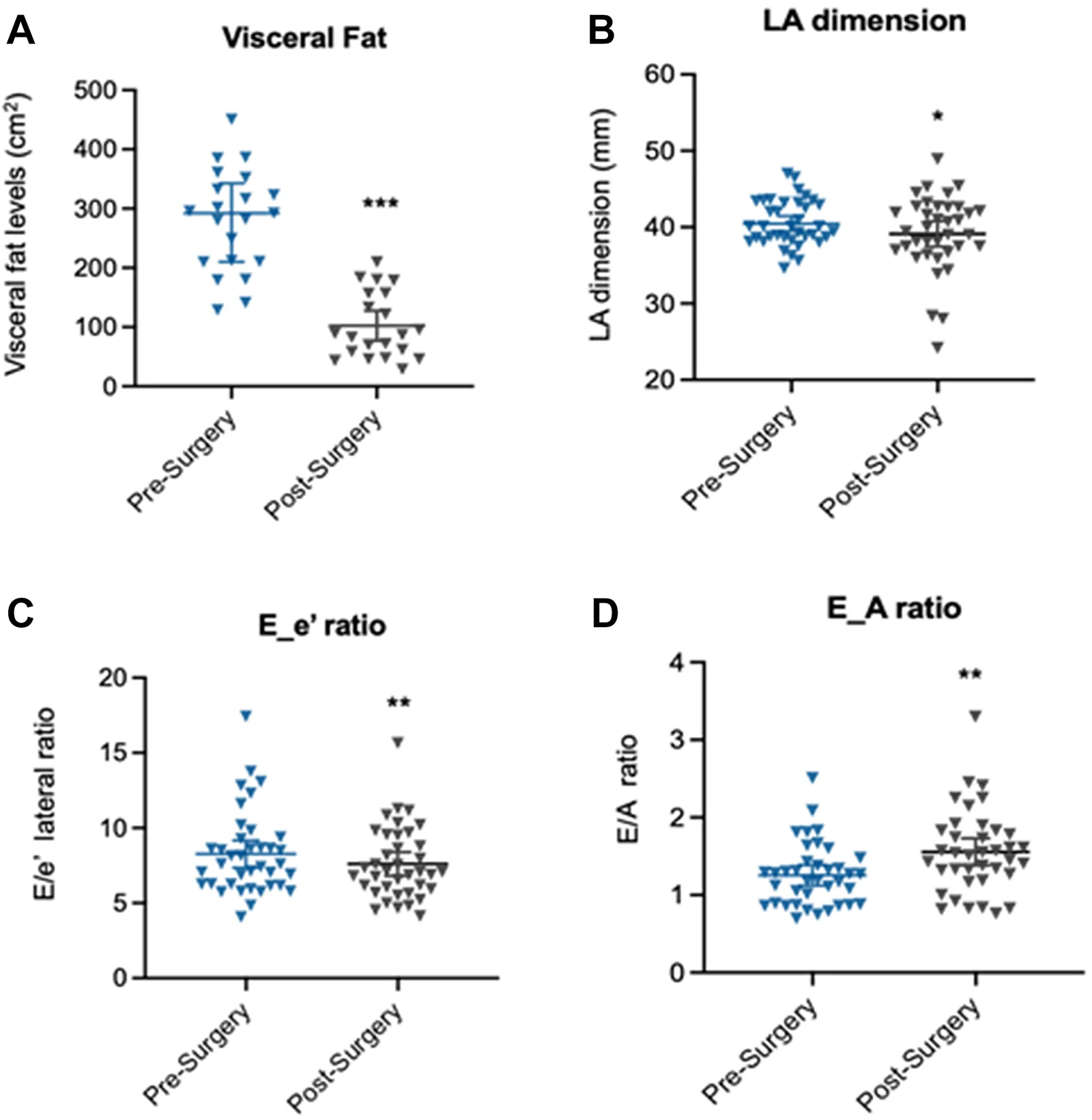
Visceral Adiposity and Diastolic Function Following Bariatric Surgery Visceral fat levels (n = 21) (A), left atrial dimension (B), lateral E/e’ ratio (C), and E/A ratio (D), in the preoperative period and at 12 months after bariatric surgery. The boxes are presented with median (central line), percentiles 25 (lower line) and 75 (upper line). Whiskers represent the upper and lower adjacent values. ∗ <0.05, ∗∗ <0.01, ∗∗∗< 0.001 compared with the presurgery. LA = left atrial.

**Figure 4:**
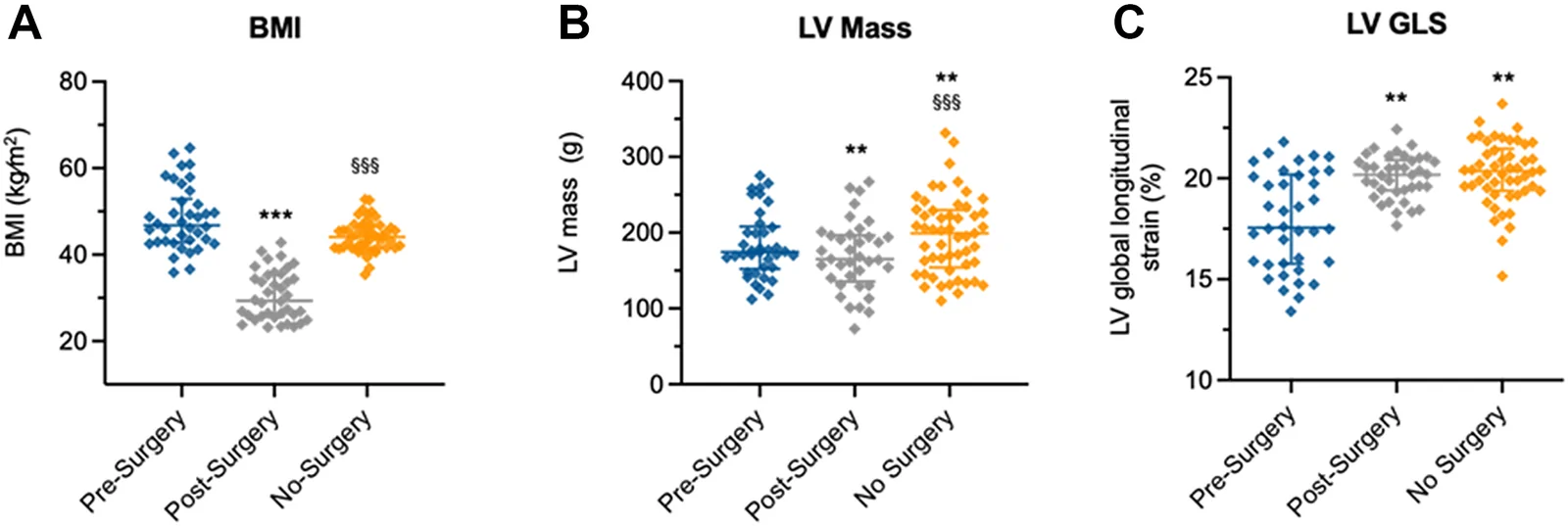
Global Adiposity, Cardiac Remodeling, and Function Following Bariatric Surgery Body mass index (A), left ventricular mass (B) and left ventricular global longitudinal strain (C) in the preoperative period (presurgery), at 12 months after bariatric surgery (postsurgery) vs obese controls (no surgery). The boxes are presented with median (central line), percentiles 25 (lower line) and 75 (upper line). Whiskers represent the upper and lower adjacent values. ∗ <0.05, ∗∗ <0.01, ∗∗∗<0.001 compared with the preoperative surgical group. § < 0.05, §§ < 0.01, §§§ <0.001 compared with the postoperative surgical group. LV = left ventricular; BMI = body m index.

After 1 year, surgical treatment of obesity led to favorable change in LV diastolic function performance, with a 25% enhance E/A ratio, a decrease in left atrial dimension, and reduction in lateral E/e' ratio (8.3 ± 2.8 to 7.6 ± 2.5) and septal E/e' ratio (10.8 ± 3.3 to 9.8 ± 2.5) (all *P* < 0.05) (Figure 3). The prevalence of abnormal LV diastolic function was significantly lower at 12-month follow-up (42% vs 24%). Systolic parameters were also improved during follow-up. LV subclinical cardiac function improved following surgery, evidenced by an increase in LV GLS postoperatively (16.3% ± 2.5% vs 18.8% ± 1.3%, *P* < 0.05), and the proportion of patients with abnormal LV subclinical cardiac function (LV GLS <18%) was reduced by 70% at 1-year (Figure 1, Figure 4). LV ejection fraction remained normal during follow-up. RV function (RV Tapse) was significantly improved after surgery. Others parameters of RV function (RV free wall strain and RV GLS) tended all to improve. NT-proBNP level increased from 50 ± 7 to 147 ± 18 pg/mL (*P* < 0.01). The subclinical myocardial function deteriorated at follow-up in 38% of participants with obesity managed conservatively.

### Clinical determinants of cardiac remodeling and function after bariatric surgery

Improvement in LV subclinical cardiac function following bariatric surgery was positively correlated with changes in BMI, percentage of weight loss, fat mass, visceral adipose tissue levels, insulin resistance, inflammatory markers, and postoperative BMI (all *P* < 0.05). No significant correlation was observed with age, sex, subcutaneous adipose tissue levels, blood pressure, glucose and lipid measures, heart rate variability parameters, NT-proBNP levels, preoperative type 2 diabetes, hypertension, LV hypertrophy, or LV diastolic dysfunction status (all *P* > 0.05). After adjusting for clinical relevant covariates (percentage of weight loss, changes in BMI, visceral adipose tissue levels, insulin resistance, and inflammatory markers), only change in BMI (R^2^-adjusted 43.3, *P* = 0.001) and inflammatory markers (high-sensitivity C- reactive protein) (R^2^-adjusted 12.7, *P* = 0.04) remained independent predictor of changes in subclinical cardiac function (LV GLS). No significant contribution of age, heart rate, blood pressure, preoperative hypertension, LV diastolic function, or LV hypertrophy was observed in the final model. When preoperative diabetes was forced into the multivariate models, the results remained unchanged. In multivariable regression analyses, a postoperative LV subclinical cardiac function within normal (LV GLS ≥18%) was associated with favorable changes in systolic blood pressure, visceral fat levels (*P* = 0.05), and positive change in heart rate variability parameters. Changes in visceral adiposity, insulin resistance, and type 2 diabetes status were independent predictors of favorable change in LV diastolic function. Reduction in visceral adipose tissue levels was the only independent predictor of LV hypertrophy regression (R^2^-adjusted 36.8, *P* = 0.004).

### Subgroup analysis according to adiposity mobilization after bariatric surgery

Recovery from LV cardiac abnormalities after bariatric surgery was evaluated according to global adiposity and visceral adiposity mobilization. We found significant improvement in LV subclinical myocardial function, regardless of postoperative adiposity categories based on BMI. However, abnormalities of LV subclinical cardiac function (LV GLS) in the follow-up were more frequent in individuals who remained obese (47% participants with BMI ≥30 kg/m^2^) (obese vs nonobese, 17.9% ± 1.9% vs 19.1% ± 1.6%, *P* = 0.05). In addition, majority of adults with abnormal postoperative LV diastolic function (76%) were characterized by residual obesity (BMI ≥30 kg/m^2^). Additional analysis on postoperative visceral adiposity mobilization (median change of visceral adipose tissue) showed lower prevalence of LV hypertrophy and LV concentric remodeling, and greater reduction in left atrial dimension (35 vs 41 mm, *P* < 0.05) with higher visceral fat reduction. Comparable benefit on LV cardiac function following surgery was observed between both subgroups (*P* < 0.05).

## Discussion

The main findings of the present study are: 1) surgical treatment of obesity reversed multiple obesity-related metabolic conditions/comorbidities involved in HF development; 2) additional benefits on cardiac remodeling and parameters of diastolic and systolic cardiac function were observed, even if half of participants remained in the obesity BMI category range at 1 year; 3) a greater weight loss and achieving a BMI <30 kg/m^2^ after bariatric surgery resulted in greater benefits on cardiac remodeling and function; 4) visceral adipose tissue mobilization mediated part of cardiac benefits; 5) conversely, a substantial proportion of patients with obesity managed medically worsen adverse cardiac remodeling and function during follow-up.

### Effect of bariatric surgery on cardiac remodeling and indexes of diastolic and systolic cardiac function

Bariatric surgery is currently the only treatment option associated with substantial and durable weight reduction in individuals with severe obesity, in whom there is a markedly increased incidence of HF.^29^ Although no large prospective, randomized trial is available, several smaller cardiac imaging studies suggest that bariatric surgery has favorable effect on LV cardiac structure and function.^3^^,^^29^ Favorable changes in cardiac structure and function at 2 years following gastric bypass surgery was reported.^7^^,^^30^ Surgical treatment of obesity is associated with a significantly lower risk of HF in prospective studies with long-term follow-up.^10^^,^^11^^,^^31^ We previously demonstrated that bariatric surgery (BPD-DS), was associated with favorable cardiac remodeling and improvement in LV cardiac function shortly (6 months) following surgery.^9^ Recently, improvement in LV GLS (from 17 preoperatively to 20%) was reported 10 months after gastric bypass in a study of 40 patients (73% female gender).^30^ In the present study, we evaluated the long-term effect of the most effective metabolic surgery (ie, BPD-DS) on LV cardiac remodeling and function considering that the nadir of weight loss is reached at 12 to 18 months.

Despite younger age (mean age 41 years), asymptomatic abnormalities of cardiac remodeling and function (LV and RV myocardial strain) were frequent preoperatively in our surgery group with severe obesity and preserved LV ejection fraction. Bariatric surgery reverses early cardiac structural and functional abnormalities, as well as obesity coexisting metabolic conditions. Abnormalities of LV subclinical cardiac function (myocardial strain) were normalized in 70% of patients after surgery, with a major reduction in the prevalence of abnormal subclinical LV cardiac function (from 79% to 32%). We also show potential to improve RV systolic function (RV strain) after bariatric surgery, suggesting the high potential of bariatric surgery as a treatment for biventricular dysfunction in prevention of future HF. In a similar way, Sorimachi et al^32^ demonstrated improvement in biventricular cardiac strain after different types of bariatric surgery including 13% of BPD-DS. To our knowledge, our study is the largest study having evaluated LV and RV cardiac strain, visceral adiposity and heart rate variability parameters following BPD-DS. Most of LV diastolic function parameters were also significantly improved at 1-year, especially indexes of LV filling pressures. LV ejection fraction remained normal throughout the study, confirming its low sensitivity in detecting asymptomatic cardiac dysfunction in this high-risk population. Taken together, these results show that surgical treatment of obesity exerts important cardioprotective effects in adults with severe obesity through favorable LV and RV cardiac remodeling and LV hypertrophy regression, reversal of LV diastolic dysfunction, reduction of left atrial dimension and improvement in subclinical cardiac function (LV and RV strain). These results demonstrated the high potential of bariatric surgery as a valuable strategy for HF prevention and regression. Additional benefits may be observed on atrial fibrillation burden and atrial fibrillation prevention^33^ as we observed a decrease in abdominal visceral adiposity and left atrial size. In a large population-based study of 1,664 patients with obesity and HF who underwent bariatric surgery,^8^ risk of HF exacerbation was significantly reduced (40% reduction) and rate of emergency visit or hospitalization for HF exacerbation was significantly lower 2 years after bariatric surgery among individuals with HF. Abnormalities of LV cardiac structure and function despite a preserved LV ejection fraction should be considered as an obesity-related comorbidity that warrants earlier access to bariatric surgery in prevention of future HF.

### Potential determinants of cardiovascular benefits after bariatric surgery

The mechanisms explaining benefits of bariatric surgery on cardiac remodeling and function are unclear but likely involve a combination of multiple pathophysiological mechanisms. In this study, we explored potential determinants of this improvement using a vast comprehensive set of cardiometabolic data. We also had the advantage of studying the most effective surgery in terms of metabolic recovery.^12^ We found that cardiovascular benefits of bariatric surgery on cardiac remodeling and function were observed independently of comorbidities burden and remission. Visceral adiposity mobilization and residual obesity emerged as important mediators of favorable cardiac remodeling, and improvement in indices of diastolic cardiac function following bariatric surgery. Increased visceral adiposity is significantly associated with adverse cardiac remodeling, diastolic cardiac dysfunction, and HF development, independently of cardiovascular risk factors.^34^ Importantly, although 47% of our patients were still obese following bariatric surgery (mean BMI 30.6 ± 5.7 kg/m^2^), subclinical cardiac functions return to normal and indices of diastolic cardiac function were improved in more than half of patients. Moreover, visceral fat mass reduction (63% reduction) was the only independent predictor of LV hypertrophy regression after surgery. In a small study (26 bariatric patients, 14 gastric bypass), Rayner et al^35^ have shown that gastric bypass results in a significant reduction in visceral fat (62% reduction), and reversal of cardiac remodeling (magnetic resonance imaging) after surgery (854 ± 383 days in follow-up time). Altogether, this suggests that as long as visceral adiposity is reduced, a return to normal BMI after surgery is not necessary regarding the benefits on cardiac structure and function. The functional quality of adipose tissue depots over time may also represent an important determinant of cardiac recovery. In addition, there may be effects of bariatric surgery on obesity related proinflammatory state and altered cardiac substrates metabolism and cardiac energetics, which may influence cardiac remodeling and function.^36^ We found a significant improvement of insulin resistance, inflammatory biomarkers, and heart rate variability following bariatric surgery. Hannukainen et al.^37^ found abnormalities of cardiac remodeling and cardiac substrate metabolism (cardiac positron emission tomography) in obesity, which were reversible 6 months after gastric bypass. Future mechanistic studies are required to identify determinants of cardiovascular recovery following bariatric surgery, and among different surgical procedures. Furthermore, longitudinal studies with cardiac imaging will help to clarify whether metabolic recovery and comorbidities remission following bariatric surgery will have favorable effect in the longer term on cardiac remodeling and function.

### Study limitations

This study has several limitations. First, baseline BMI and clinical characteristics, both probably associated with the primary outcomes, differed significantly between treatment groups, and participants with higher BMI levels and higher burden of comorbidities were more likely to undergo bariatric surgery. Accordingly, selection bias and confounding by indication might have reduced the internal validity of the study. The population sample size may have limited our ability to detect significant associations between cardiac remodeling and function with others clinical and metabolic parameters. Our study contained a relatively large proportion of women, which may have influenced the observed relationship between bariatric surgery and cardiovascular benefits. However, HF with preserved ejection fraction is found to be higher in women which emphasizes the importance of studies including a majority of women. It should be noted that glucagon-like peptid-1 (GLP-1) receptor agonists, which have recently become a cornerstone of obesity management, were not widely available at the time of this study. As such, the effects of these pharmacologic therapies on cardiac remodeling and function were not assessed, and our findings reflect outcomes in the context of standard care prior to their adoption. Also, the inclusion of objective functional assessments (eg, exercise testing) would have strengthened the study by allowing better translation of the observed imaging improvements into clinically meaningful functional and outcome-related benefits.

Notwithstanding these limitations, our study has several strengths. It was conducted in the largest sample of patients who undergone the most efficient metabolic surgery with a standardized cardiac imaging assessment of RV and LV subclinical cardiac function using speckle tracking echocardiography. We also used CT imaging–derived gold standard measurements of visceral fat levels, heart rate variability parameters, and a large comprehensive set of clinical and metabolic variables. Finally, the ability to analyze longitudinal measures of subclinical cardiac function following bariatric surgery in relation with metabolic recovery provide new insights and perspective in a growing comorbid population with increased risk of HF.

## Conclusions

Surgical treatment of obesity is associated with reversal of subclinical abnormalities of cardiac remodeling and function and might exert a possible cardioprotective effect on HF incidence (Central Illustration). Bariatric surgery could be effective in prevention and remission of HF, which may have a substantial impact on HF burden with obesity. Larger prospective studies are warranted to confirm these observations and determine their long-term clinical implications.

Perspectives **COMPETENCY IN MEDICAL KNOWLEDGE:** This is the first study demonstrating that surgical management of obesity (biliopancreatic diversion with duodenal switch) reverses subclinical abnormalities of cardiac remodeling and function in adults with obesity and preserved left ventricular ejection fraction. These results support the high potential of bariatric surgery as a valuable strategy to prevent or reverse heart failure in high-risk forms of obesity.

**TRANSLATIONAL OUTLOOK 1:** Additional longitudinal and mechanistic studies are needed to determine whether improvements in cardiac structure and function following bariatric surgery last over time and translate into better cardiovascular outcomes, and to disentangle the factors underlying cardiac recovery across different procedures.

**Central Illustration:**
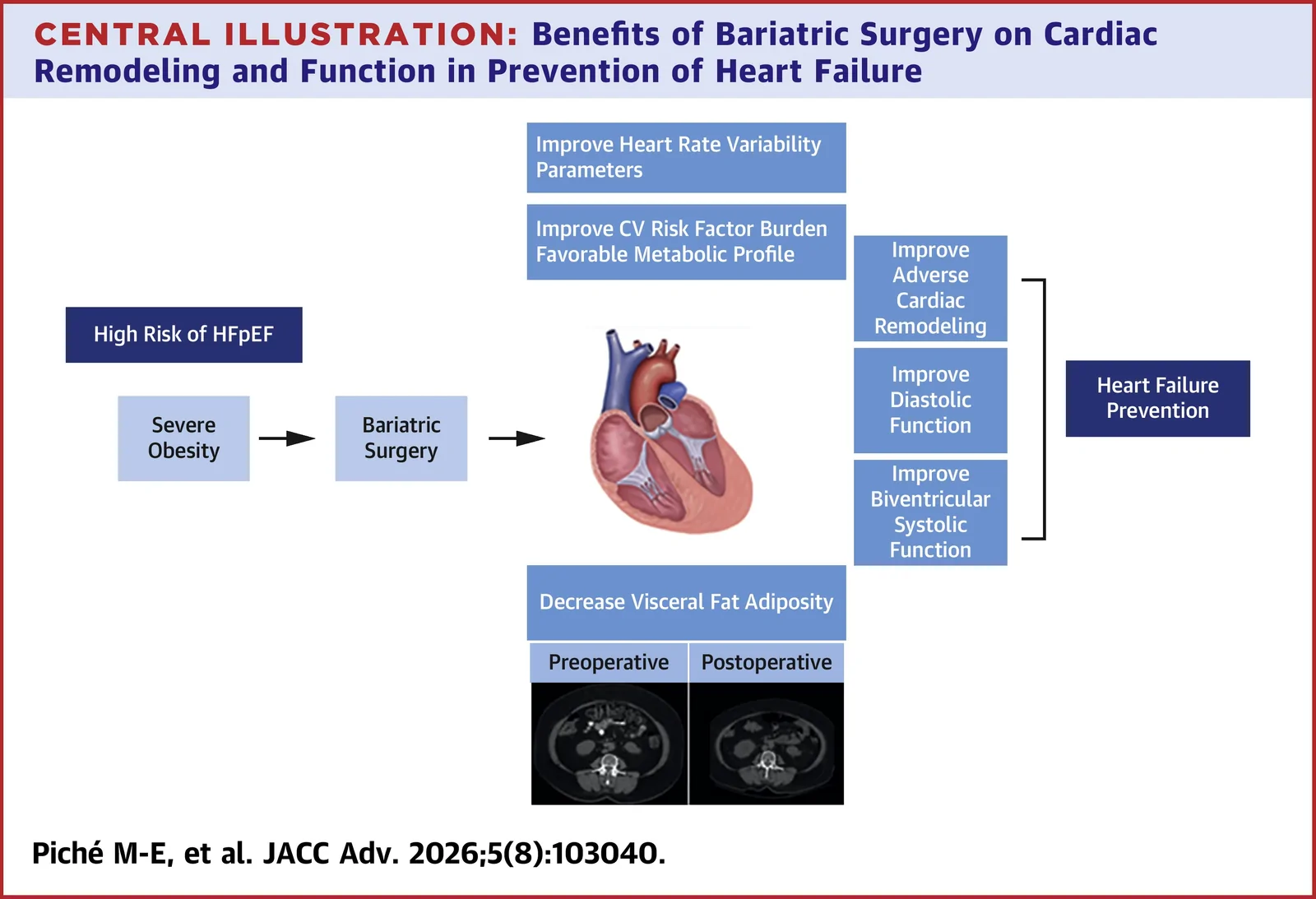
Benefits of Bariatric Surgery on Cardiac Remodeling and Function in Prevention of Heart Failure This figure shows that surgical treatment of obesity (biliopancreatic diversion with duodenal switch) exerts important cardioprotective effects in adults with severe obesity through reduction in visceral adiposity, and a favorable effect on metabolic conditions, heart rate variability parameters, cardiac remodeling (left ventricular hypertrophy regression, reduction of left atrial dimension), diastolic function, and improvement in biventricular systolic function (left and right ventricular strain). These results demonstrate the high potential of surgical treatment of obesity as a valuable strategy for heart failure prevention and regression. CV = cardiovascular; HFpEF = heart failure with preserved ejection fraction.

## Funding support and author disclosures

This work was supported by the Quebec Heart and Lung Institute Foundation. Dr Piché has received a career clinician-research award from the Fonds de Recherche du Québec-Santé (FRQS). Paul Poirier is a senior clinician-researcher from the 10.13039/501100000156FRQS. All other authors have reported that they have no relationships relevant to the contents of this paper to disclose.
