# Supplementary material for: Bariatric Surgery Improves Cardiac Remodeling and Function in Adults With Preserved Left Ventricular Ejection Fraction
Source: JACC Adv. 2026 Aug 26;5(8):103040. doi: 10.1016/j.jacadv.2026.103040 (PMC13531616; doi:10.1016/j.jacadv.2026.103040)
Supplement: Supplementary file 1 [file mmc1.docx]

**Supplemental Table 1** Heart rate variability variables

|  | | **Baseline** | **12 Months *** |
| --- | --- | --- | --- |
| Age, years | | 41 ± 11 | - |
| Female, n (%) | | 34 (89) | - |
| BMI, kg/m^2^ | | 48.4 ± 7.4 | 30.6 ± 5.7 |
| Resting Heart Rate, bpm | | 80 ± 11 | 62 ± 9 |
| Standard deviation  of the RR interval (SDNN), ms | | 112 ± 33 | 144 ± 29 |
| Standard deviation of  the mean RR calculated over a 5-minute period (SDNN index), ms | | 101 ± 31 | 125 ± 27 |
| NN50 divided by total number of NN intervals (pNN50), % | | 8 ± 1 | 17 ± 2 |
| High-frequency (HF), ms^2^ | | 153 ± 22 | 335 ± 48 |
| Low-frequency (LF), high-frequency (HF) ratio, LF/HF ratio | | 2.9 ± 0.2 | 2.5 ± 0.9 |

Data are expressed as mean ± SD or as number (percentage).

Significantly different within surgical group, * p<0.001

Abbreviations: HF, high-frequency power [0.15-0.40 Hz]; LF, low-frequency power [0.04-0.15 Hz]; SDNN: standard deviation of normal-to-normal intervals; SDNN index: SDNN index, mean of the standard deviations of all the NN intervals for each 5-min segment of a 24-h heart rate variability recording.

LV Hypertrophy

Abnormal systolic function

% of participants

§

§

§

Abnormal diastolic function

Medical group (follow-up)

Medical group (baseline)

Surgery group (follow-up)

Surgery group (baseline)

*

*

*

**Supplemental Figure 1. Changes in cardiac remodeling and function during follow-up.**

CAPTION Graph bars represent the proportion of adults with A) left ventricular hypertrophy, B) abnormal diastolic function, and C) abnormal systolic function (abnormal [LV GLS <18%]). preoperative (blue) and postoperative (grey) * < 0.05, ** < 0.01, ***<0.001. LV: left ventricular.
